# DIAROP: Automated Deep Learning-Based Diagnostic Tool for Retinopathy of Prematurity

**DOI:** 10.3390/diagnostics11112034

**Published:** 2021-11-03

**Authors:** Omneya Attallah

**Affiliations:** Department of Electronics and Communications Engineering, College of Engineering and Technology, Arab Academy for Science, Technology and Maritime Transport, Alexandria 1029, Egypt; o.attallah@aast.edu

**Keywords:** Retinopathy of Prematurity (ROP), Deep Learning (DL), transfer learning, Convolutional Neural Networks (CNN), Computer-Aided Diagnosis

## Abstract

Retinopathy of Prematurity (ROP) affects preterm neonates and could cause blindness. Deep Learning (DL) can assist ophthalmologists in the diagnosis of ROP. This paper proposes an automated and reliable diagnostic tool based on DL techniques called DIAROP to support the ophthalmologic diagnosis of ROP. It extracts significant features by first obtaining spatial features from the four Convolution Neural Networks (CNNs) DL techniques using transfer learning and then applying Fast Walsh Hadamard Transform (FWHT) to integrate these features. Moreover, DIAROP explores the best-integrated features extracted from the CNNs that influence its diagnostic capability. The results of DIAROP indicate that DIAROP achieved an accuracy of 93.2% and an area under receiving operating characteristic curve (AUC) of 0.98. Furthermore, DIAROP performance is compared with recent ROP diagnostic tools. Its promising performance shows that DIAROP may assist the ophthalmologic diagnosis of ROP.

## 1. Introduction

Retinopathy of Prematurity (ROP) impacts preterm infants and could cause blindness. Screening and Diagnosing procedures of ROP have several barriers in developing countries [1]. Initially, there are inadequate amounts of medical screening/imaging equipment for ROP. Also, the number of staff for airing retinal images for ROP is relatively small. Moreover, ophthalmologists’ training procedure is not consistent, and the qualified ophthalmologists are of few numbers. In addition, the application of the ROP screening policy is insufficient in developing countries. Thus, in developing countries, numerous premature neonates become blind because of the absence of early screening and timely treatments [2]. Another obstacle that occurs worldwide is that imaging of the infants’ retina is very hard and limited due to the shortage of equipment that offers a fast and simple scanning process, specially designed for uncooperative and unanesthetized cases like premature neonates [3]. However, the latest imaging instrumentation advancements have considerably enhanced and facilitated the capacity to attain high-quality images from premature infants.

The enhancement in digital imaging delivered new strategies for the diagnosis, monitoring, and treatments of ROP. Though the typical binocular indirect ophthalmoscopy (BIO) is considered the gold standard for imaging infants with ROP, it needs proper and extensive training by qualified and skilled staff. Currently, the wide-angle digital retinal imaging (Retcam) system is extensively used for examining premature neonates with ROP. This is because it is simpler and faster to function compared to BIO. Moreover, the Retcam system can capture, store, and transmit fundus images from several angles. Additionally, it is more favorable for medical examination and monitoring, education, and scientific research. All these benefits confirm acceptable results when using it along with digital image analysis techniques to diagnose ROP [4].

In recent years, several diagnostic tools based on artificial intelligence (AI) techniques have been proposed to diagnose medical conditions [5]. Examples of such conditions are as cancer [6,7,8,9], eye abnormalities [10,11], brain tumors and mental disorders [12,13], heart problems [14,15,16,17,18], gastrointestinal diseases [19], motor disabilities [20,21], and lung diseases [22,23]. With the latest advancements in digital imaging of ROP such as Retcam, fundus retinal images may be analyzed effectively with AI methods. The AI methods include traditional machine learning (ML) and modern Deep Learning approaches (DL). In the former methods, conventional image processing and feature extraction techniques identify pathological patterns such as fundus lesions and blood vessels [24]. Also, they involve manual outlining of chosen features, and hence systemic bias can occur from handcrafted extraction [4]. On the other hand, the DL approaches do not need any image processing or feature extraction steps for performing the diagnosis [25], Therefore, they are preferred over ML approaches. Moreover, diagnostics tools based on DL have numerous advantages over manual diagnosis. First, they are effective and faster than manual diagnosis. In addition, they are easier and prevent the error caused by fatigue or emotions [26]. Also, they are more accurate, especially with large data. For these reasons, this paper aims to propose a robust and reliable diagnostic tool called DIAROP based on DL techniques for the automatic diagnosis of ROP in its early stages with high accuracy. This tool is constructed using fundus images for preterm infants acquired using Retcam. It uses an ensemble of DL techniques to perform classification. DIAROP has the potential to help ophthalmologists in the early and accurate diagnosis of ROP. The study also aims to present a diagnostic tool to differentiate normal images and those with ROP. DIAROP can reduce the time and labor caused by manual diagnosis and conventional ML approaches.

The key contributions of this study are as follows:

1. Four state-of-the-art pre-trained CNNs models of different architectures are investigated; these pre-trained CNNs varied in their convolutional layers amount and principal building block.
2. The performance of the four pre-trained models is compared. Considerable alteration in performance is noticed.
3. Features extracted from these networks are of high dimension, so they are reduced using Fast Walsh Hadamard transform (FWHT).
4. Due to the difference in performance across the different CNNs, feature integration is utilized to merge each of the CNN architecture’s benefits.
5. Integrated features extracted from the four pre-trained CNNs are examined to select the integrated features set with the highest impact.
6. Feature integration is achieved using three fusion techniques, including auto-encoder (AE), principal component analysis (PCA), and discrete wavelet transform (DWT), to investigate the best technique which improves the performance.

This paper is organized as follows; Section 2 illustrates the previous diagnostic tools for ROP diagnosis. Section 3 presents the methodology and materials. It also introduces the description of DIAROP; the proposed diagnostic tool. Section 4 provides the performance metrics and parameter settings. Section 5 delivers the results of DIAROP. Section 6 introduces a discussion of the main settings and results of DIAROP. Section 7 concludes the paper.

## 2. Previous Diagnostic Tools

Several ROP diagnostic tools based on Retcam imaging modality have been proposed in the literature during the past few years based on traditional machine learning techniques (TML) or Deep Learning techniques (DL). The criteria behind the selection of the related works are based on recent studies that focused on diagnosing only ROP. In other words, those studies built diagnostic tools for identifying diabetic retinopathy for premature infants, not adults or children. Regarding TML, the authors of Ref. [27] employed several ML classifiers to diagnose pre-plus, plus, and non-plus ROP disease using 87 images. They achieved an average accuracy of 80.15%. In 2018, an ROP tool was constructed using 20 images to differentiate between pre-plus and plus ROP diseases [28]. Oloumi et al. utilized Gabor filters to distinguish between plus and non-plus ROP disease using 110 images of 41 patients [24,29]. Ataer-Cansizoglu et al. [30] proposed a diagnostic tool called “i-ROP” to classify healthy, pre-plus and plus ROP disease based on 77 images. They employed a support vector machine (SVM) classifier and achieved an accuracy of 95%. Nevertheless, the previous tools used low-quality images, a small number of images to construct the classification model. They required manual feature extraction and segmentation of the vessels, which might reduce the accuracy of diagnosis because of the probable faults and professional bias that could occur while choosing the target vessels. Also, the time needed for image processing like segmentation and feature extraction is high. This necessitate the need for a more automated tools that can be reliable such as those based on DL techniques.

Lately, numerous ROP diagnostic tools based on DL techniques have been introduced. These systems utilized transfer learning (TL) which reemploys pre-trained Convolutional Neural Networks (CNNs) trained with large datasets such as ImageNet on another similar classification problem but with a lower number of images like the one on hand [31]. Since the pre-trained CNN has been previously learned image features from a massive dataset with many diverse images, TL has been proven to enhance diagnostic accuracy [32,33,34]. Brown et al. [35] proposed a tool called “i-ROP “ based on two CNNs, the first one for segmentation and the second for classifying healthy, pre-plus and plus ROP disease. The authors used 5511 retinal images and attained an average sensitivity of 96.5% and an average specificity of 94%. Another automated system based on DL was presented called “DeepROP” [36]. The authors employed 11,707 images. The system first diagnosed images into normal or ROP, and then ROP images were classified into severe or minor. The system achieved a 96.64% sensitivity, 99.33% specificity for classifying normal versus ROP, and an 88.46% sensitivity, 92.31% specificity for severe versus minor. An automatic tool was introduced called “ROP.AI” to diagnose normal and plus diseases [37].The classification model achieved 96.6% sensitivity and 98.0% specificity. Lei et al. [38] employed ResNet-50 and added an attention module and channel to diagnose ROP. Similarly, Zhang et al. [39] employed several versions of ResNet to build their model. On the other hand, Rani et al. [40] introduced a tool based on DL and multiple instance learning (MIL) where images were split into equal patches, and then a CNN was employed to extract features from these patches. Features of the same image are combined to distinguish between normal and ROP cases. The system obtained an accuracy of diagnosis of 83.33%, sensitivity and specificity of 100%, and 71.43%, correspondingly. Later the authors of [41] proposed a pipeline called “I-ROP ASSIST” to differentiate among healthy and plus ROP diseases. The authors segmented the images using U-Net CNN and extracted handcrafted features from these segmented images to train several machine learning classifiers. The highest accuracy achieved was 94%.

Huang et al. [42] presented an automatic tool based on five CNNs, including VGG-13, VGG-16, MobileNet, Inception, and DenseNet to detect ROP in preterm infants. The authors first distinguished between normal and ROP cases and then classified ROP severity into mild and severe. As described in [43,44], the several stages of ROP were categorized as Stage 1, Stage 2, and Stage 3. The ophthalmologists classified the severity of ROP as mild-ROP, in case the eye condition refers to Stage 1 and Stage 2 ROP. Otherwise, if the eye condition refers to Stage 3 ROP, then it is a severe ROP. The authors compared the performance of the 5 CNNs and showed that VGG-16 has the highest performance. For normal versus ROP, the VGG-16 reached 96% accuracy, 96.6% sensitivity, and 95.2% specificity. For severity classification, the VGG-16 model attained 98.82% accuracy, 100% sensitivity, and 98.41% specificity. Similarly, in Ref. [45] an automated model was constructed based on VGG-16, ResNet-50, and Inception CNNs. The model first recognized ROP cases and then classified their severity into mild and severe. Maximum accuracy of 97% was achieved using the Inception CNN for healthy versus ROP and 84% for mild versus severe. Another automated system based on DL called “DeepDR” was proposed in [46]. The system employed an ensemble of CNNs to identify ROP and then its severity. DeepDR consists of two fusion stages; the first stage is feature-based fusion, whereas the second is a probability-based fusion. The results showed that fusing the average probabilities of Xception, Inception, and InceptionResNet CNNs attained the highest sensitivity of 97.5%, a specificity of 97.7% for identifying ROP. While identifying the severity level, the same CNNs achieved a sensitivity of 98.1% and a specificity of 98.9%. The results achieved by diagnostic tools based on DL have shown that these tools are comparable and can produce higher accuracy than experts in diagnosing ROP disease. Therefore, they have the potential to be employed for ROP diagnosis [4]. However, they have some limitations; the majority of the previous techniques were based on only spatial DL features, however, merging spatial DL features with other types of features such as spectral or temporal can boost the accuracy of image classification [47,48,49,50]. Besides, almost all of them utilized a single convolution neural network (CNN) to perform classification, but it was proven that fusing features of several CNN are capable of enhancing the classification performance [51,52,53,54]. Furthermore, they employed private datasets which makes it difficult to use these datasets for comparison purposes and reproducing their works. This study proposed an automated diagnostic tool called “DIAROP” based on DL techniques. It can be considered as a reliable diagnostic tool that can classify ROP with high accuracy using a large number of images. It consists of ensembles of DL approaches that are fused using several integration techniques. DIAROP utilizes TL to four convolution neural networks (CNNs) and compares their performance for diagnosing ROP. These CNNs include ResNet-50, Inception, Inception-ResNet, and Xception. It is assessed on a public freely available dataset.

## 3. Materials and Methods

### 3.1. ROP Data Acquisition

**Figure 1:**
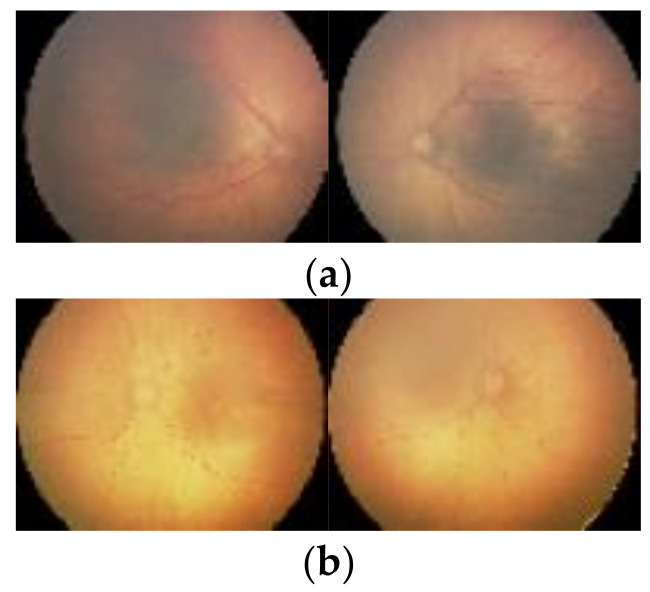
Samples of images included in the dataset, (**a**) Diseased, (**b**) Not Diseased.

The ROP images collected were a part of the ROP Collaboration Group (RCG), which includes 30 hospitals located all over China. Shenzhen Eye Hospital (SEH) was the leader for this collaboration as it offers ROP screening facilities to all other hospitals participating in this collaboration. All these hospitals applied similar ROP screening standards for selecting the cases involved in the dataset collection. The initial number of infants who were asked to join the study was 26,424. However, many of them were omitted from the acquisition process. Only neonates who were eligible to participate should have the following two criteria. First, they should have a birth weight lower than 2000 g. Second, premature neonates who are 2000 g at birth but suffer from severe systemic disorders. The eligible infants were around 890. Every premature infant’s eye (each eye) was captured with ten standard angles (10 images) throughout each screening process. the number of generated images was 8090 for ROP cases and 9711 for normal eyes. The images were acquired using Retcam 2 or Retcam 3 by an expert technician Five professional childhood ophthalmologists independently annotated the images acquired during the screening procedure to “Disease” and “Not Diseased”. In the case of inconstancies, the final decision was taken after a discussion made by the ophthalmologists’ group. The dataset contains 8090 diseased images and 9711 not diseased images. More details about the data acquisition process can be found in [26]. Samples of the dataset’s images are shown in Figure 1.

### 3.2. Proposed Diagnostic Tool

**Figure 2:**
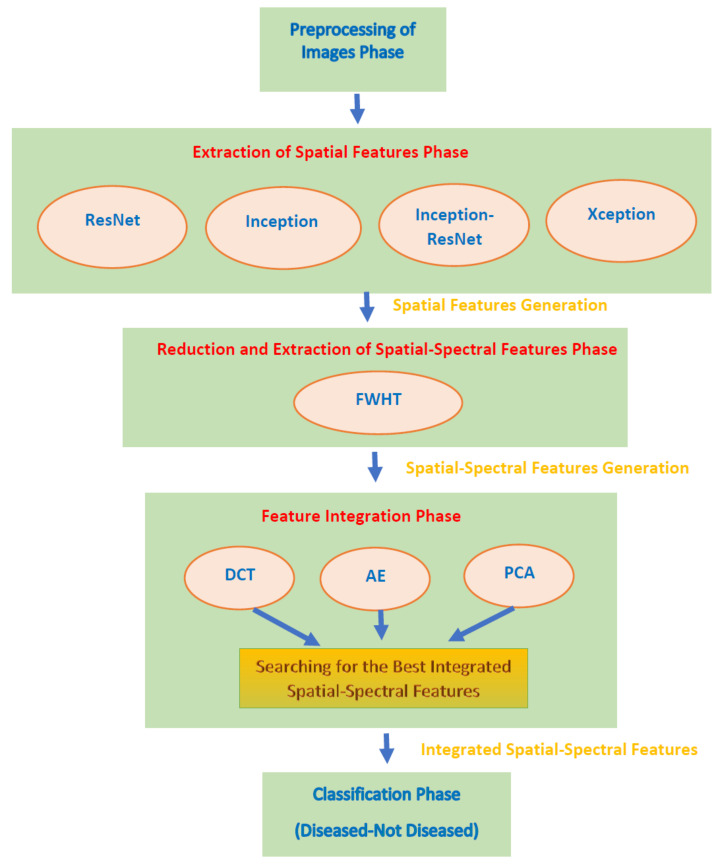
The five phases of the DIAROP diagnostic tool.

This study proposes an automated diagnostic tool named DIAROP for the early diagnosis of ROP. It is based on an ensemble of DL techniques and three integration approaches. DIAROP consists of five phases involving, the pre-processing of images, extraction of spatial features, reduction and extraction of spatial-spectral features, integration of features, and classification phases. Initially, images are resized and augmented in the first phase (pre-processing of images phase). Afterward, four CNNs, including; ResNet-50, Inception V3, Xception, and Inception-ResNet V2, are utilized for pulling out spatial DL features using TL in the second phase. Next, spectral features are extracted using Fast Walsh Hadamard transform (FWHT) in the third phase, which ends up by generating spatial-spectral features. The features’ dimension is also reduced in this phase. Then, three integration techniques are employed to combine features comprising; auto-encoder (AE), principal component analysis (PCA), and discrete wavelet transform (DWT). Finally, in the classification phase, three well-known machine learning classifiers including, linear support vector machine (L-SVM), Quadratic-SVM (Q-SVM), and Linear discriminate analysis (LDA) classifiers are used to classify diseased and not diseased cases. The five phases of DIAROP are illustrated in Figure 2.

#### 3.2.1. Pre-Processing of Images Phase

ROP images are resized to be equivalent to the input layer’s dimension of the four CNNs used in DIAROP. For the Inception V3, Xception, and Inception-ResNet V2 CNNs, the input size is 229 × 229 × 3. Whereas for ResNet-50 the input size is 224 × 224 × 3. Next, these images are augmented using translation (−30,30), scaling (0.9,1.1), flipping in x and y directions, shearing (0,45) in x and y directions. Augmentation is usually done to expand the number of fundus images in a dataset and prevent overfitting [55,56].

#### 3.2.2. Extraction of Spatial Features Phase

TL is used in this phase to adapt four pre-trained CNNs that were previously trained on the ImageNet dataset to classify ROP disease. These pre-trained CNNs involve Inception V3, ResNet-50, Xception, and Inception-ResNet V2. Next, the output layers of these pre-trained CNNs are modified to be equal to 2, equivalent to the number of classes of the ROP dataset instead of the 1000 classes of ImageNet. Afterward, a few parameters are altered (illustrated later in the parameter selection section), and then the CNNs are trained. TL is also applied to obtain spatial features from a particular layer of each CNN. These features are taken from the “avg_pool” of ResNet-50, Xception, InceptionResNet, and Inception CNNs. The dimension of those features after being extracted is 2048 for Inception, Xception, and ResNet-50. Whereas for Inception-ResNet V2, the number of features is 1536.

#### 3.2.3. Reduction and Extraction of Spatial-Spectral Features Phase

In this phase, FWHT is utilized to extract spectral features from the spatial DL features attained in the previous phase from the four CNNs resulting in obtaining spatial-spectral features. FWHT is a practical and time-efficient approach to calculate the Walsh Hadamard Transform (WHT), which analyzes and transforms the input data into a cluster of perpendicular waveforms termed Walsh basis functions having coefficients of 1 and −1. It is considered one of the simplest transforms as it is based on additions and subtractions operations. It analyzes the input data of length 2^n^ into 2n coefficients, equivalent to the discrete WHT of the input data. The fundamental privilege of FWHT is that it simple, fast, and involves small storage capacity space to save the decomposed coefficients. It decomposes the input data with a dimension of power 2, but if the power is lower than 2, its dimension is padded with zero to become equal to the subsequent closer power of 2. [57,58]. FWHT is also used for data reduction. Thus, it is also applied to reduce the spatial features’ dimension extracted in the preceding phase forming reduced spatial-spectral features.

#### 3.2.4. Feature Integration Phase

This phase integrates the reduced spatial-spectral features obtained from the four CNNs after applying FWHT in the previous phase. This integration is made using three integration techniques, including PCA, AE, and DWT. These three approaches are also employed to reduce the vast dimension of features occurring due to the feature integration process. This phase also explores the best set of integrated spatial-spectral features that impacts the classification performance. This investigation is made by, first, exploring the integration of each two reduced spatial-spectral featured attained from every two CNNs. Second, it examines the integration of every three reduced spatial-spectral featured extracted from every three CNNs. Finally, it observes the integration of all reduced spatial-spectral featured extracted from the four CNNs. The three integration techniques are briefly discussed below.

**PCA** is a method that is frequently used to lower the massive size of observed variables in a dataset. It performs covariance analysis on the observed variables to compress the data and remove redundancy. It transforms the original variables into new transformed functions of lower dimensions. These functions are called principal components, which represent the variance located in the original data. PCA is commonly used when the data is enormous [59].

**AE** is an unsupervised DL technique commonly used to decrease the massive data dimension [60]. AE involves two core elements, encoder, and decoder. The former maps the observed features into a code utilizing a hidden layer that illustrates a code applied to characterize the input and enable the network to neglect redundant information. The latter element maps the code to recreate the original input demonstration of the data.

**DWT** applies orthogonal basis functions termed wavelets to analyze input data [61]. For 1-D input data as the spatial-spectral features, the DWT procedure is accomplished through convolving the input features with a low and high pass filter [62]. After That, a reduction process is accomplished by downsampling the output data by 2 [63]. Subsequently, two clusters of coefficients are produced called the approximation coefficients CA1 and detail coefficients CD1 [64]. In this study, the mother wavelet utilized is the Meyer wavelet (dmey). For reducing the dimension of the integrated spatial-spectral features, only CD1 coefficients are employed.

#### 3.2.5. Classification Phase

The classification phase of DIAROP is used to diagnose ROP and classify images as either diseased or not diseased. This classification is done using three well-known machine learning classifiers involves, Linear-SVM (L-SVM), Quadratic SVM (Q-SVM), and Linear discriminant analysis (LDA). 5-fold cross-validation is employed to validate the results. Note the classification phase is accomplished through three settings corresponding to the extraction of spatial features, reduction and extraction of spatial-spectral features, and feature integration phases of DIAROP. In setting I, spatial features extracted from each CNNs are used separately to train the three classifiers. Whereas in setting II, each spatial-spectral feature set obtained from each CNN is used individually to learn the classifiers. Finally, in setting III, the integrated feature sets generated in the feature integration phase are utilized for training the classifiers.

## 4. Performance Setting

### 4.1. Parameters Adjustment

For training the four CNNs, some parameters are tuned while others are kept unchanged. The hyper-parameters adjusted in the CNNS are the minibatch size which is the number of samples included in each sub-epoch weight change and is chosen to be 10. This number was chosen to fits the memory of the GPU, as increasing it lead to an “out of memory” problem. Utilizing small batch sizes generally reaches the greatest generalization performance [65]. The learning rate defines the stride size at every iteration whilst turning on the way to a minimum of a loss function. It was selected to be 0.0003 which achieved the highest accuracy while minimizing the training time. It is well-known that increasing the epochs size increases the training time. The sum of epochs was modified to 10 as rising this number did not enhance the performance. The validation frequency is chosen to be 1246 to calculate the accuracy only once by the end of each epoch. The learning technique used for training the four CNNs is stochastic gradient descent with momentum. The three settings of DIAROP are accomplished using Matlab 2020 a. The processor is Intel(R) Core (TM) i7-10750H with NVIDIA GeForce GTX 1660 video controller of 6 GB capacity, processor frequency of 2.6 GHz 64-bit operating system.

### 4.2. Metrics of Assessment

The assessment metrics used to evaluate the performance of the three settings of DIAROP are illustrated in this section. These metrics involve sensitivity, accuracy, specificity, F1-score, and precision. These metrics are measured utilizing the following mathematical functions [66] (1)–(5).

Sensitivity is also known as Recall or true positive rate which is equivalent to the number of diseased images that were correctly classified over the total number of true diseased images.

(1) $$Sensitivity=\frac{TP}{TP+FN}\;$$

Accuracy is a metric that calculates the number of images that were correctly classified by the classifier.

(2) $$Accuracy=\frac{TP+TN}{TN+FP+FN+TP}\;$$

Specificity is equivalent to the number of non-diseases images that were correctly classified over the total number of true non-diseases images.

(3) $$Specificity=\frac{TN}{TN+FP}\;$$

F1-score is the average of precision and recall metrics commonly employed to access the performance. Its value varies from ‘0’ to ‘1’. If the score is ‘0’ this means extremely bad performance. The higher the F1 score, the better the performance.

(4) $$F1-Score=\frac{2\times TP}{\left(2\times TP\right)+FP+FN}\;$$

Precision is a well-known metric that determines the ratio of correctly classified diseased images over the total number of images classified as diseased.

(5) $$Precision=\frac{TP}{TP+FP}\;$$

where; True positive (*TP*) is the total number of correctly diagnosed images as diseased. *TN*. True negative is the total number of correctly diagnosed images as not diseased. False-positive (*FP*) is the sum of images that were incorrectly diagnosed as diseased. False-negative is the sum of images that were inaccurately diagnosed as not diseased.

## 5. Results

**Figure 3:**
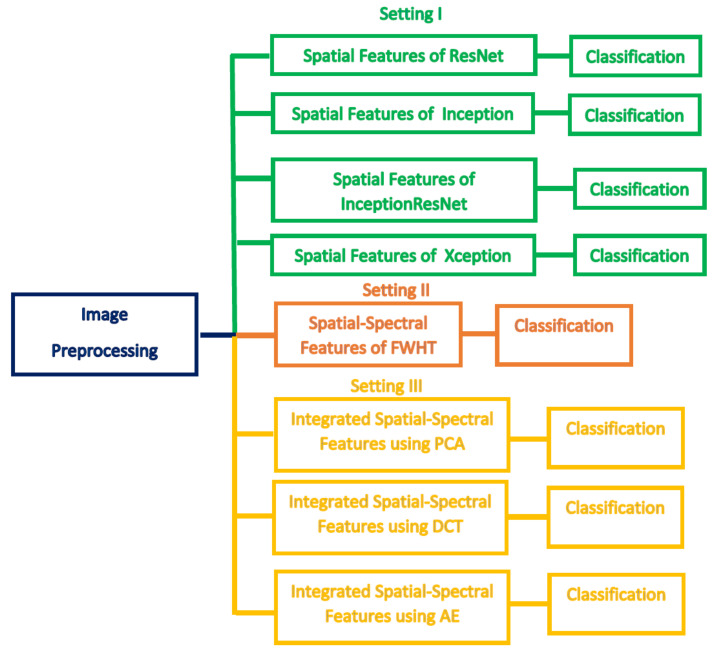
The three classification settings of DIAROP diagnostic tool.

This section illustrates the results of the three settings of the DIAROP diagnostic tool. In the first setting, spatial features obtained from the four CNNs are employed to train the three classifiers individually. Next, in the second setting of DIAROP, the spatial-spectral features extracted and reduced using FWHT are utilized to learn the classifiers. In the third setting, the sets of integrated features generated in the feature integration phase are employed to train the classifiers. Figure 3 represents the three classification settings of DIAROP.

### 5.1. Setting I Results

The results of the spatial features obtained from the four CNNs and employed to train the three classifiers are shown in Table 1. As can be noticed from Table 1, the highest accuracies of 90.9%, 91.6%, and 91.6% are achieved using the LDA, L-SVM, and Q-SVM classifiers trained with ResNet-50 spatial features. The next highest performance is attained utilizing the spatial features of Inception-ResNet V2, where the accuracies are equal to 90.2%, 90.6%, and 90.8% using the LDA, L-SVM, and Q-SVM classifiers. The following are the spatial features of Inception V3, where the accuracies obtained are 89.4%, 90.5%, and 90.5% using the LDA, L-SVM, and Q-SVM classifiers. The spatial features of Xception CNN attain the lowest accuracy of 87.3%, 88.3%, and 88.6% using the LDA, L-SVM, and Q-SVM classifiers.

### 5.2. Setting II Results

**Figure 4:**
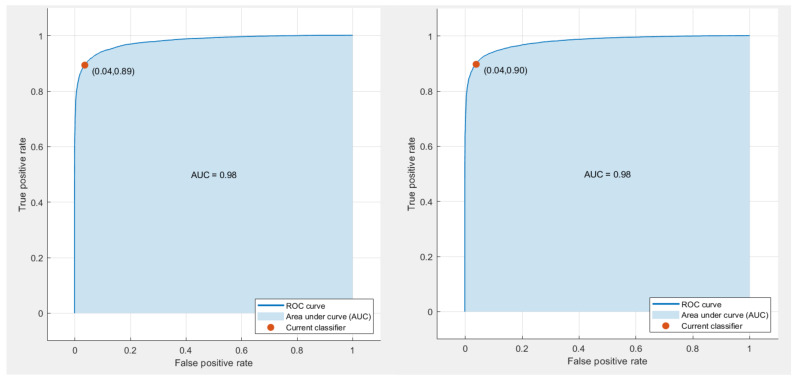
ROC curve analysis and AUC for (**Left**), L-SVM, (**Right**) Q-SVM trained with the spatial-spectral features involving ResNet50+Inception+InceptionResNet integrated using DWT.

The reduced spatial-spectral features obtained after applying the FWHT method are discussed in this section. The accuracy attained using the four spatial-spectral features used to construct the three classifiers are displayed in Table 2. Maximum accuracies of 91.1%, 91.6%, and 91.8% are attained using spatial-spectral features of ResNet-50. The spatial-spectral features of Inception-ResNet V2 reach the next maximum accuracies of 90.5%, 90.6%, 90.8% using the LDA, L-SVM, and Q-SVM classifiers. Then, the spatial-spectral features of Inception V3 attain accuracies of 89.9%, 90.6%, and 90.6% for the LDA, L-SVM, and Q-SVM classifiers. Finally, the least accuracies of 87.8%, 88.5%, and 88.8% are achieved using the spatial-spectral features of Xception. It can be observed from Table 2 that spatial-spectral obtained using FWHT has enhanced the accuracy of the LDA, L-SVM, and Q-SVM classifiers compared to those obtained in Table 1. This is not the case for the L-SVM classifier trained with spatial-spectral features of Xception. Note that for the L-SVM and Q-SVM classifiers constructed with spatial-spectral features of Inception-ResNet V2, the accuracies are the same as setting I as shown in Figure 4. However, the number of features has been reduced from 1536 (setting I) to 700 in setting II. Similarly, the number of spatial-spectral features obtained from ResNet-50, Inception, and Xception is decreased from 2048 in setting I to 1100 in setting II.

### 5.3. Setting III Results

The results of the feature integration are discussed in this section. Three integration techniques, including DWT, AE, and PCA, are compared. Initially, every two spatial-spectral features obtained from FWHT are integrated. Similarly, every three spatial-spectral features are integrated. Finally, the whole spatial-spectral features are fused. The results of the feature integration phase are illustrated in Table 3, Table 4 and Table 5. Table 3 shows the accuracies of the three classifiers trained with the integrated spatial-spectral features fused using AE. In the case of integrating two spatial-spectral features, the peak accuracies of 92%, 92.6%,92.6%, and 92%, 92.5%, 92.4% are achieved by LDA, L-SVM, and Q-SVM classifiers. These classifiers are trained with ResNet50+Inception and ResNet50+InceptionResNet spatial-spectral features respectively. Whereas for integrating three spatial-spectral feature sets, the maximum accuracy of 92.6%, 93%, and 93.1% is obtained using the LDA, L-SVM, and Q-SVM classifiers. These classifiers are learned with the spatial-spectral features of ResNet-50+Inception+InceptionResNet.

On the other hand, integrating the four spatial-spectral features reached an accuracy of 92.5%, 92.8%, and 93% using the LDA, L-SVM, and Q-SVM classifiers. It was proven from Table 3, that integrating the three spatial-spectral features of ResNet-50+Inception+InceptionResNet using AE has the highest performance amongst all other integrated feature sets fused These results verify that integrating spatial-spectral features obtained from different CNNs can improve the diagnostic capability of DIAROP.

The results of spatial-spectral integration using PCA are displayed in Table 4. When investigating the integration of every two spatial-spectral features, it was found that fusing the spatial-spectral features of ResNet-50+Inception and ResNet-50+Inception-ResNet has attained the highest accuracy. These accuracies are 92.2%, 92.6%, 92.6% and 92.4%, 92.5%, 92.6% utilizing LDA, L-SVM, and Q-SVM classifiers respectively. While examining the combination of every three spatial-spectral features, the peak accuracy of 92.7%, 92.9%, and 92.9% is reached using LDA, L-SVM, and Q-SVM classifiers. These models are built with the spatial-spectral features of ResNet50+Inception+InceptionResNet. Nevertheless, the maximum accuracies among all integrated feature sets are accomplished using the LDA, L-SVM, and Q-SVM classifiers (92.8%, 92.8%, and 93.1%) developed using the spatial-spectral features of the four CNNs of DIAROP. These performances confirm that integrating spatial-spectral features from several CNNs can enhance the performance of DIAROP.

The accuracy attained by the DWT approach as an integration technique is shown in Table 5. The results in Table 5 show that for fusing every two spatial-spectral features, the highest accuracies of 92%, 92.6%, and 92.7% are achieved with LDA, L-SVM, and Q-SVM classifiers. These classifiers are constructed using ResNet50+InceptionResNet. On the other hand, when fusing every three feature sets, the maximum accuracies are attained using the LDA, L-SVM, and Q-SVM models. These models are constructed with the spatial-spectral features of ResNet50+Inception+InceptionResNet. Accuracies of 92.4% and 93.2% are obtained using the LDA and Q-SVM classifiers trained with the spatial-spectral features of the four CNNs. Where a higher accuracy of 93.2% is attained using the L-SVM classifier trained with the spatial-spectral features of the four CNNs. These accuracies prove that feature integration is capable of increasing the capability of DIAROP in diagnosing ROP.

A comparison between the highest accuracy and the number of features achieved using each feature integration technique is shown in Table 6. The previous results showed that the integrated spatial-spectral features of the ResNet50+Inception+InceptionResNet fused using the three integration techniques achieved the highest performance using the Q-SVM classifier. The table indicates that the three integration techniques have comparable performance; however, the highest performance is achieved using DWT. Table 6 also indicates that DWT that obtained the highest accuracy has 1500 features, followed by the AE with 1800 features, ending with PCA with only 300 features.

The performance metrics are calculated for the spatial-spectral features of ResNet50+Inception+InceptionResNet integrated using DWT and displayed in Table 7. The table shows that the sensitivities of 88.31%, 89.4%, 89.7%, specificities of 95.88, 96.4%, 96.1%, precisions of 94.7%, 95.4%, 95.1%, and F1-scores of 91.39%, 92.3%, 92.3% are attained using the LDA, L-SVM, and Q-SVM respectively. Figure 4 shows the receiving area characteristics curve (ROC) and the area under the ROC curve (AUC) for the L-SVM and Q-SVM classifiers. These models are trained with the spatial-spectral features involving ResNet-50+Inception+InceptionResNet integrated using DWT. Figure 4 indicates that the AUC for L-SVM and Q-SVM is 0.98. As mentioned in [67,68], for a diagnostic tool to be reliable, it should attain a precision and specificity greater than 95% and a sensitivity of more than 80%. Thus, with the performance metrics of DIAROP shown in Table 7, DIAROP is considered a reliable diagnostic tool that can be used for the automatic diagnosis of ROP.

## 6. Discussion

**Figure 5:**
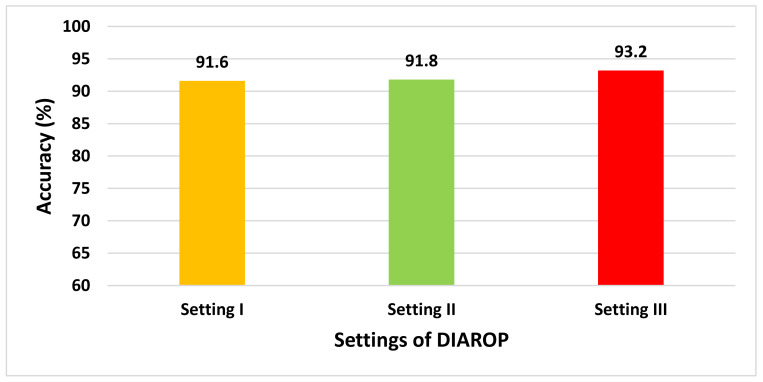
The accuracy (%) attained at the three settings of DIAROP.

**Figure 6:**
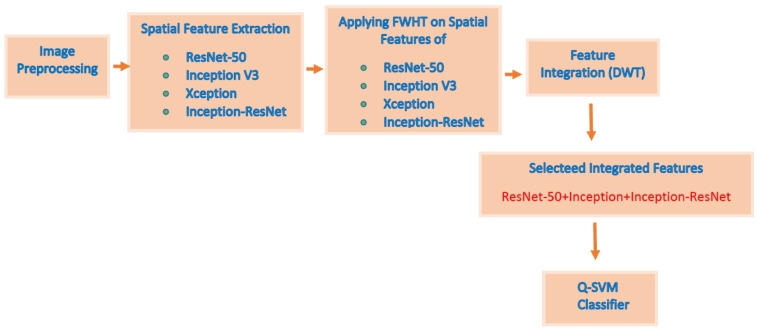
The final architecture of DIAROP diagnostic tool.

This paper proposed an automated diagnostic tool called DIAROP to diagnose ROP based on DL techniques. It consists of several DL approaches based on TL. DIAROP involves three settings. In the first setting, TL is used to obtain spatial features from four pre-trained CNNs. These spatial features are utilized separately to train LDA, L-SVM, and Q-SVM classifiers. Setting II, presents the extraction of spectral features from the spatial features obtained from each CNN. These spectral features are extracted using FWHT after applying it to the previous setting’s spatial features, ending up producing spatial-spectral features. FWHT is also utilized to reduce these spatial-spectral features’ dimensions. The last setting presents the feature integration phase using three integration techniques. It searches for the best integrated spatial-spectral features extracted from the four CNNs. Figure 5 shows the highest accuracy attained in each setting. In setting I, an accuracy of 91.6% was obtained using the spatial features of ResNet-50. On the other hand, an accuracy of 91.8% was achieved using the spatial-spectral features of ResNet-50. Finally, in the last setting, the spatial-spectral features of ResNet50+Inception+InceptionResNet integrated using DWT attained an accuracy of 93.2% using the Q-SVM classifier. The final architecture of DIAROP is displayed in Figure 6.

The training time achieved by DIAROP is compared with those attained by end-to-end DL CNNs, including ResNet-50, Inception V3, Xception, and Inception-ResNet V2. The training execution times of DIAROP and end-to-end DL CNNs are illustrated in Table 8. This table proves the competence of DIAROP as the training execution time of DIAROP is 404.17 s, much lower than that executed by other end-to-end DL CNNs.

Combining several features extracted from different Deep Learning models, increase the complexity and the computational time of the classification process. However, the proposed DIAROP diagnostic tool tried to reduce this side effect by performing two feature reduction steps. The first is using the FWHT to reduce the dimension of the spatial Deep Learning feature extracted from each CNN. Next, it employs several integration techniques which are also well-known feature reduction methods such as PCA, AE, and DWT. It can be noted in the results that they have reduced the number of features as shown in Table 7 and reduced the classification time compared to end-to-end models as shown in Table 8.

Regarding the CNNs employed in this study, they were chosen as they were previously used in the ROP’s literature such as [39,45,46] and achieved good performance. For feature extraction, TL has been employed as it is a well-known technique commonly used in several ways, one of the most ways is to use it to extract deep features from specific layers of a CNN [33,69]. On the other hand, the FWHT is a common technique used for dimensionality reduction [57]. It is also a well-known method for time-frequency representation [58]. The results section has proved that FWHT has successfully reduced the number of features while enhancing performance. (Please refer to Table 1 and Table 2). PCA [70], AE [71], and DWT [72,73] techniques are employed for the integration process as they are popular feature reduction methods that have been extensively used in the machine learning literature. SVM performs well with large dimension space and as it uses kernel function which maps the feature space into a new domain that can easily separate between classes of a dataset. Therefore, it is commonly used with the huge dimension of DL features extracted from CNNs [74] achieving outperforming results.

The main challenge of this study is the availability of ROP datasets. To our knowledge, all datasets that have been used in the literature are private; therefore, it was hard to compare the performance of DIAROP with related previous diagnostic tools. The only article that used the same dataset employed to construct DIAROP is [26], where the authors used three CNNs individually to construct their diagnostic tool. In contrast, DIAROP integrates the spatial-spectral features of several CNNs instead of using only one CNN individually. The performance of DIAROP is compared with the AlexNet, VGG-16, and GoogleNet CNNs’ performance, as shown in Table 9. Also, it was compared with other end-to-end models used in the literature. The table confirms the competitiveness of DIAROP over the other methods, as the accuracy (93.2%) of DIAROP is higher than the accuracy of the AlexNet (77.9%), VGG-16 (80.4%,), and GoogleNet (73.9%) used in [26]. The outperformance of DIAROP over end-to-end CNNs as the accuracy accomplished using DIAROP is 93.2% which is higher than the 86.5%, 90.9%, 91.42%, and 91.48% achieved using Xception, Inception-ResNet V2, Inception V3, and ResNet-50 CNNs. The outperformance of DIAROP ensures that it can be used as a diagnostic tool that helps ophthalmologists diagnose the ROP disease more accurately. It is a reliable automated tool that can lower examination time and ophthalmologists’ exertion in the diagnosis procedure.

It was proven in several studies the great training capacity of Deep Learning with large datasets without the necessity to identify significant features by professionals, to automatically diagnose ROP disease in retinal scans accurately [36]. Deep Learning could deliver similar diagnostic results on a given retinal scan each time, which is hard to be achieved by ophthalmologists as the diagnosis achieved by them is subjective. The key findings of this study are: (1) DIAROP can assist ophthalmic experts to achieve high ROP diagnostic accuracy avoiding challenges of the manual diagnosis; (2) the diagnosis achieved by DIAROP is not subjective; (3) DIAROP offers a new vision regarding the diagnostic procedures for ophthalmologists, particularly those who are located in developing and poor countries, and finally (4) ophthalmologists in the area of ROP are limited which can affect the diagnosis procedure, however, DIAROP can help in the timely diagnosis of the ROP disease, that could help in lowering blindness caused by the misdiagnosis. This can attain a major contribution in the field of ophthalmology research, clinical, and educational applications. It may also help ophthalmologists in choosing suitable follow-up plans according to the diagnostic results.

## 7. Conclusions

This study introduced an automated diagnostic tool named DIAROP based on an ensemble of DL techniques to diagnose ROP disease. DIAROP involved five phases, including pre-processing of ROP images, extraction of spatial features, reduction and extraction of spatial-spectral features, integration of features, and classification phases. These phases were performed in three different settings. Setting I presents the mining of spatial DL features from four pre-trained CNNs using TL. Whereas setting II describes the extraction of spatial-spectral features using FWHT after being operated on the spatial features of setting I. FWHT is also applied to reduce the extracted features’ size. Finally, in the last setting, the feature integration process is performed where each combination of the integrated feature set is investigated to select the best set of integrated spatial-spectral features extracted from several CNNs. The results of setting II showed that using spatial-spectral features is better than using spatial features only. The reason is that setting II accuracy is higher than setting I. Also, the number of features in setting II is lower than setting I. Moreover, setting III’s results proved that the integrated spatial-spectral features of several CNNs could enhance ROP disease’s diagnostic accuracy. The results were compared with end-to-end DL CNNs and a recent related diagnostic tool that verified the competence of DIAROP. Therefore, DIAROP is considered a robust and reliable diagnostic tool that can automatically diagnose ROP disease with high accuracy. DIAROP can reduce the manual labor and the time of examination accomplished during the diagnosis procedure. The limitations of this study are; first, it did not apply segmentation techniques. Also, it did not consider categorizing the severity of the ROP disease. Moreover, the real ROP diagnosis includes Aggressive posterior ROP, acute ROP, plus disease ROP, pre-threshold ROP, and ROP Zones and stages that were not addressed in the paper. Future work will address these limitations. Future work will also consider using more CNNs. Furthermore, upcoming work will apply DIAROP to detect the severity of the ROP disease.

## Figures and Tables

**Table 1:** The accuracy (%) attained using spatial features extracted from the four CNNs of DIAROP diagnostic tool.

Spatial Features	LDA	L- SVM	Q-SVM
**ResNet-50**	90.9	91.6	91.6
**Inception**	89.4	90.5	90.5
**Xception**	87.3	88.7	88.6
**InceptionRes**	90.2	90.6	90.8

**Table 2:** The accuracy (%) attained using spatial-spectral features obtained after applying FWHT on the spatial features extracted from the four CNNs of DIAROP diagnostic tool.

Spatial-Spectral Features	LDA	L-SVM	Q-SVM
**FWHT-ResNet-50**	91.1	91.6	91.8
**FWHT-Inception**	89.9	90.6	90.6
**FWHT-Xception**	87.8	88.5	88.8
**FWHT-InceptionResNet**	90.5	90.6	90.8

**Table 3:** The accuracy (%) attained for the three classifiers trained with integrated spatial-spectral features using AE.

Integrated Spatial-Spectral Features	LDA	L-SVM	Q-SVM
**Integration of Two Spatial-Spectral Feature Sets**			
**ResNet50+Inception**	92	92.6	92.6
**ResNet50+Xception**	91.3	91.8	91.9
**ResNet50+InceptionResNet**	92	92.5	92.4
**Inception+Xception**	90.5	90.7	90.9
**Inception+InceptionResNet**	91.5	91.8	91.6
**Xception+InceptionResNet**	91	91.3	91
**Integration of Three Spatial-Spectral Feature Sets**			
**ResNet-50+Inception+Xception**	92.1	92.7	92.6
**ResNet-50+Inception+InceptionResNet**	92.6	93	93.1
**ResNet-50+InceptionResNet+Xception**	92.3	92.7	92.9
**Inception+InceptionResNet+Xception**	91.6	92.1	92.3
**Integration of Four Spatial-Spectral Feature sets**			
**All**	92.5	92.8	93

**Table 4:** The accuracy (%) achieved for the three classifiers trained with integrated spatial-spectral features using PCA.

Integrated Spatial-Spectral Features	LDA	L-SVM	Q-SVM
**Integration of Two Spatial-Spectral Feature Sets**			
**ResNet50+Inception**	92.2	92.6	92.6
**ResNet50+Xception**	91.8	92.2	92.3
**ResNet+InceptionRes**	92.4	92.5	92.6
**Inception+Xception**	91.1	91.2	91.1
**Inception+InceptionResNet**	91.8	91.9	91.8
**Xception+InceptionResNet**	91.3	91.3	91.4
**Integration of Three Spatial-Spectral Feature Sets**			
**ResNet50+Inception+Xception**	92.6	92.5	92.7
**ResNet50+Inception+InceptionResNet**	92.7	92.9	92.9
**ResNet50+Xception+InceptionResNet**	92.5	92.6	92.8
**Inception+Xception+InceptionResNet**	92.1	92.3	92.2
**Integration of Four Spatial-Spectral Feature Sets**			
**All**	92.8	92.8	93.1

**Table 5:** The accuracy (%) achieved for the three classifiers trained with integrated spatial-spectral features using DWT.

Integrated Spatial-Spectral Features	LDA	L-SVM	Q-SVM
**Integration of Two Spatial-Spectral Feature Sets**			
**ResNet50+Inception**	91.9	92.6	92.6
**ResNet50+Xception**	91.4	92.2	92.3
**ResNet50+InceptionResNet**	92	92.6	92.7
**Inception+Xception**	90.7	91.4	91.5
**Inception+InceptionResNet**	91.5	92.2	92.1
**Xception+InceptionResNet**	91	91.4	91.4
**Integration of Three Spatial-Spectral Feature Sets**			
**ResNet50+Inception+Xception**	92.1	92.9	92.9
**ResNet50+Inception+InceptionResNet**	92.4	93	93.2
**ResNet50+Xception+InceptionResNet**	92.1	92.7	92.9
**Inception+Xception+InceptionResNet**	91.7	92.3	92.1
**Integration of Four Spatial-Spectral Feature Sets**			
**All**	92.4	93.2	93.2

**Table 6:** A comparison between the highest accuracy (%) with variance and the number of features achieved using each feature integration technique.

Integration Method	Accuracy (%)	Number of Features
**AE**	93.1 (0.003)	1800
**PCA**	92.9 (0.187)	300
**DWT**	93.2 (0.13)	1500

**Table 7:** The Performance Metrics (%) for the spatial-spectral features of ResNet50+Inception+InceptionResNet integrated using DWT.

Classifier	Sensitivity	Specificity	Precision	F1-Score
**LDA**	88.31	95.88	94.7	91.39
**L-SVM**	89.4	96.4	95.4	92.3
**Q-SVM**	89.7	96.1	95.1	92.3

**Table 8:** The training execution time (sec) of the four CNNs and DIAROP.

Method	Training Time (Seconds)
**Xception**	11,410
**Inception-ResNet V2**	30,426
**Inception V3**	16,221
**ResNet-50**	4276
**DIAROP**	404.17

**Table 9:** A comparison between DIAROP and recent work based on the same dataset.

Reference	Method	Accuracy (%)
[26]	**AlexNet**	77.9
[26]	**VGG-16**	80.4
[26]	**GoogleNet**	73.9
[46]	**Xception**	86.95
[46]	**Inception-ResNet V2**	90.9
[45]	**Inception V3**	91.42
[39]	**ResNet-50**	91.48
	**DIAROP**	93.2

## Data Availability

The data presented in this study are openly available in [IEEE Dataport].

## Abbreviations

AE	Auto-encoder
AI	Artificial intelligence
BIO	Binocular indirect ophthalmoscopy
CA	Approximation coefficients
CD	Details coefficients
CNN	Convolutional Neural Network
DL	Deep Learning
dmey	Meyer wavelet
FN	False-negative
FP	False-positive
DWT	Discrete wavelet transform
FWHT	Fast Walsh Hadamard transform
LDA	Linear discriminate analysis
L-SVM	Linear support vector machine
ML	Machine learning
MIL	Multiple instance learning
Q-SVM	Quadratic support vector machine
RCG	ROP Collaboration Group
Retcam	Wide-angle digital retinal imaging
ROP	Retinopathy of Prematurity
PCA	Principal component analysis
SEH	Shenzhen Eye Hospital
SVM	Support vector machine
TL	Transfer learning
TN	True negative
TR	True positive
TML	Traditional machine learning techniques
WHT	Walsh Hadamard Transform
